# Oxidized Phospholipids Induce Ceramide Accumulation in RAW 264.7 Macrophages: Role of Ceramide Synthases

**DOI:** 10.1371/journal.pone.0070002

**Published:** 2013-07-31

**Authors:** Lingaraju M. Halasiddappa, Harald Koefeler, Anthony H. Futerman, Albin Hermetter

**Affiliations:** 1 Institute of Biochemistry, Graz University of Technology, Graz, Austria; 2 Core Facility for Mass Spectrometry, Medical University of Graz, Graz, Austria; 3 Department of Biological Chemistry, Weizmann Institute of Science, Rehovot, Israel; Temple University, United States of America

## Abstract

Oxidized phospholipids (OxPLs), including 1-palmitoyl-2-glutaroyl-*sn*-glycero-3-phosphocholine (PGPC) and 1-palmitoyl-2-oxovaleroyl-*sn*-glycero-3-phosphocholine (POVPC) are among several biologically active derivatives that are generated during oxidation of low-density lipoproteins (LDLs). These OxPLs are factors contributing to pro-atherogenic effects of oxidized LDLs (OxLDLs), including inflammation, proliferation and death of vascular cells. OxLDL also elicits formation of the lipid messenger ceramide (Cer) which plays a pivotal role in apoptotic signaling pathways. Here we report that both PGPC and POVPC are cytotoxic to cultured macrophages and induce apoptosis in these cells which is associated with increased cellular ceramide levels after several hours. In addition, exposure of RAW 264.7 cells to POVPC and PGPC under the same conditions resulted in a significant increase in ceramide synthase activity, whereas, acid or neutral sphingomyelinase activities were not affected. PGPC is not only more toxic than POVPC, but also a more potent inducer of ceramide formation by activating a limited subset of CerS isoforms. The stimulated CerS activities are in line with the C_16_-, C_22_-, and C_24:0_-Cer species that are generated under the influence of the OxPL. Fumonisin B1, a specific inhibitor of CerS, suppressed OxPL-induced ceramide generation, demonstrating that OxPL-induced CerS activity in macrophages is responsible for the accumulation of ceramide. OxLDL elicits the same cellular ceramide and CerS effects. Thus, it is concluded that PGPC and POVPC are active components that contribute to the capacity of this lipoprotein to elevate ceramide levels in macrophages.

## Introduction

Macrophage apoptosis is a prominent feature of advanced atherosclerotic plaques. Previous studies have identified apoptotic macrophages in animal and human atherosclerotic lesions, along with other vascular cells [1,2]. There is a strong correlation between apoptotic macrophages and acute vascular events including plaque rupture, suggesting that macrophages are key determinants of plaque instability [3]. However, the significance and the factors influencing macrophage apoptosis in atherosclerosis remain poorly understood. Oxidative modification of low-density lipoproteins (LDL) is an initiating process during atherosclerosis and thought to play a critical role in oxidant injury leading to local inflammation and apoptotic events in the vascular cells [4–7]. LDL is readily oxidized at the protein and lipid moieties leading to the generation of oxidized LDL (OxLDL), which is characterized by complex mixtures of toxic oxidation products. OxPLs, including 1-palmitoyl-2-glutaroyl-*sn*-glycero-3-phosphocholine (PGPC) and 1-palmitoyl-2-oxovaleroyl-*sn*-glycero-3-phosphocholine (POVPC) are present in OxLDL and atherosclerotic lesions and are contributing factors mediating the detrimental effects of OxLDL [8]. Many studies are available explaining the mechanisms of OxLDL cytotoxicity. However, the contribution of the oxidized phospholipids (OxPLs) to these effects remains unclear. They are often associated with various stress response mechanisms including inflammatory conditions, oxidative stress, and apoptosis. Although the importance of OxPLs has been established, understanding of the initial interaction with the cells and signaling mechanisms is limited.

The sphingolipid ceramide (Cer) is a bioactive lipid mediator regulating many cellular stress response pathways under the influence of various external stimuli [9]. Various cellular and environmental stresses such as chemotherapeutics [10], ischemia-reperfusion [11], ultraviolet radiation [12] and ionizing radiation [13] can induce ceramide generation either by enzyme-catalyzed hydrolysis of complex sphingolipids, hydrolysis of sphingomyelin by sphingomyelinase (SMase), or by the acylation of the long chain sphingoid bases sphinganine and sphingosine by ceramide synthases (CerS) via *de novo* and salvage pathways, respectively. Recently, six CerS isoforms have been identified that regulate *de novo* generation of distinct ceramide species, each using a restricted set of fatty acyl-CoAs of specific chain length [14–18]. Increasing evidence suggests that different CerS isoforms are activated under the influence of various stress stimuli in a cell-specific manner. There are also reports suggesting an interplay among the various CerS such that the formed Cer species play a critical role in determining the fate of a cell under the influence of particular stress stimuli [19,20]. It has been demonstrated that POVPC and PGPC can activate caspase-3 and 8 leading to apoptotic cell death by increasing cellular ceramide levels via SMase activation [21,22]. Another study showed that OxPAPC-induced activation of nSMase/ceramide leads to the inhibition of LPS action in human aortic endothelial cells (HAEC) [23]. Perhaps surprisingly, given the pivotal roles of ceramide signaling in various stress response pathways, information is lacking on CerS-mediated Cer generation and signaling under the influence of OxPLs.

Here we report that oxidized phospholipids (OxPLs) including 1-palmitoyl-2-(5-oxovaleroyl)-sn-glycero-3-phosphocholine (POVPC) and 1-palmitoyl-2-glutaroyl-*sn*-glycero-3-phosphocholine (PGPC) stimulate Cer synthesis in RAW 264.7 macrophages by activating a limited subset of CerS isoforms. We found that PGPC is more toxic and a more potent inducer of Cer generation by specifically enhancing the activities of CerS2 and CerS5/6 in a time-dependent manner. Concomitantly, PGPC increased Cer levels significantly, especially long chain C_16_-, and very long chain C_22_-, and C_24:0_-Cer. In contrast, POVPC treatment influenced Cer levels to a lesser extent. These results provide evidence for the differential regulation of CerS isoforms by OxPLs. Our data contribute to the growing body of knowledge elucidating the specific ceramide response to stress stimuli in a cell type-specific manner.

## Materials and Methods

### Materials

Cell culture materials were obtained from Sarstedt (Numberecht, Germany) or Greiner (Kremsmunster, Austria). Dulbecco’s modified Eagle’s medium and heat-inactivated fetal bovine serum were from Invitrogen (Leek, The Netherlands). PBS and other cell culture supplements were obtained from PAA (Linz, Austria), unless otherwise indicated. Oxidized phospholipids (POVPC and PGPC) were synthesized in our laboratory or purchased from Avanti Polar Lipids (Alabaster, USA). Fumonisin B1 (FB1) was from Calbiochem (Darmstadt, Germany).

### Cell culture

The murine macrophage-like cell line RAW 264.7 (ATCC No. TIB-71, American Type Culture collection, Rockville, MD, USA) was a kind gift from Prof. Dagmar Kratky, Medical University of Graz, Austria. Cells were maintained in DMEM (4.5 g/l glucose, 25 mM HEPES, 4 mM L-glutamine, without sodium pyruvate) supplemented with 10% heat-inactivated fetal calf serum (FCS), penicillin (100 IU/mL), and streptomycin (100 µg/mL) at 37 °C in a humidified atmosphere of 5% CO_2_. Incubations with OxPLs were conducted with DMEM with or without phenol red supplemented with 0.1% FCS. Incubation mixtures were prepared by adding ethanol stock solutions of lipids to the culture medium. The ethanol concentration was below 1% (v/v) and control experiments were performed using only ethanol without lipid additives.

### Isolation and oxidative modification of LDL

Human LDL was isolated by density ultracentrifugation in OptiSeal tubes using a Beckman NVT65 Rotor at 4 ^°^C [24] from pooled healthy fresh plasma (a kind gift of Dr. Gholam Ali Khoschsorur, University Hospital, Graz). The LDL fraction was collected and stored at 4 ^°^C for up to 8 days prior to use. Lipoprotein concentration is expressed by protein measured by the method of Bradford [25]. LDL was desalted using PD 10 columns (GE Healthcare, Munich, Germany) prior to oxidative modification. Oxidation of LDL was performed by incubating with 1 mM CuSO_4_ in sterile H_2_O at 37 ^°^C for 48 h protected from light. OxLDL was desalted prior to use as described earlier.

### Cell viability assay

Cells (5 x 10^4^ Cells/well) were treated with OxPLs in 96 well plates and cellular viability was analyzed by the Vybrant® MTT cell proliferation Assay kit according to the manufacturer’s protocol. Briefly, after removal of the medium, 100 µL of MTT (5 mg/10 mL of PBS) was added to each well and incubated at 37 ^°^C for 4 h The MTT solution was removed, and 50 µL of dimethyl sulfoxide (DMSO) was added. The color intensity of the soluble formazan was measured at 570 nm after 10 minutes.

### Flow cytometric apoptosis assay

Cells (5 x 10^5^ Cells/well) were incubated for 4 h with OxPLs, washed twice in cold PBS and stained for apoptotic (labeling of surface phosphatidylserine with Alexa Fluor®488 Annexin V), necrotic (labeling of DNA in permeabilized cells with propidium iodide) and late apoptotic/early necrotic cells (Annexin V and propidium iodide labeling). Samples were then analyzed using a FACSCalibur flow cytometer (BD Bioscience, Heidelberg, Germany) and data were processed using the WINMDI 2.8 software package as described previously [26].

### RNA isolation and cDNA synthesis

Total RNA was isolated using RNeasy® mini kit (Qiagen) according to the manufacturer’s protocol. The concentration and quality of RNA samples were evaluated spectrophotometrically. Complementary DNA was synthesized from 1 µg of the total RNA using Verso cDNA kit (Thermo Scientific).

### Real-time quantitative PCR (RT-qPCR) analysis of CerS expression

RT-qPCR reactions were performed using TaqMan® Gene Expression assays and Universal PCR Master Mix using a 7300 Sequence Detection System (Applied Biosystems). Quantitative analysis was performed using a standard curve generated by serial dilutions of cDNA for each CerS gene. The relative quantitative mRNA level was determined using the comparative *Ct* method using Hprt and Gusb as the reference genes. Primer sequences were as follows (Applied Biosystems): [Mm00433562_m1 (CerS1), Mm00504086_m1 (CerS2), Mm03990709_m1 (CerS3), Mm01212479_m1 (CerS4), Mm00510991_m1 (CerS5), Mm00556165_m1 (CerS6), Mm01545399_m1 (Hprt), Mm00446953_m1 (Gusb)]. PCR reaction conditions included initial denaturation at 95 °C for 10 min, followed by 40 cycles of 15 s at 95 °C, 30 s at 56 °C and 30 s at 68 °C. The data were normalized to an internal control gene, GAPDH.

### Enzyme assays

#### In vitro ceramide synthase assay

Cells were harvested and homogenized in HEPES buffer [20 mM HEPES-KOH (pH 7.2), 25 mM KCl, 2mM MgCl_2_, and 250 mM sucrose] containing a protease inhibitor cocktail-AEBSF, 104 mM; Aprotinin, 80 µM; Bestatin, 4 mM; E-64, 1.4 mM; Leupeptin, 2 mM and Pepstatin A, 1.5 mM (SIGMA-ALDRICH). Protein concentrations were measured using the Bradford method (Bio-Rad). CerS activity was assayed as described previously [14]. Briefly, cell homogenates were incubated in a final volume of 250 µL HEPES buffer with 0.25 µCi of [4,5- ^3^H] sphinganine/15 µM sphinganine/20 µM defatted-bovine serum albumin and 50 µM of different fatty acyl-CoAs [CerS2 utilize both C_22:0_-and C_24:0_-CoAs equally as substrates. Because of solubility problems with C_24:0_–CoA, only C_22:0_–CoA was used for activity measurements] in accordance with the substrate specificity of each CerS at 37 ^°^C for 20 min. Reactions were terminated by addition of three volumes of chloroform/methanol (1/2; v/v). Lipids were extracted [27] and separated by thin layer chromatography (TLC) plate using chloroform/methanol/2M ammonium hydroxide (40/10/1; v/v/v) as solvent. [^3^H] Lipids were visualized using a phosphor imaging screen (Fuji, Tokyo, Japan), recovered from TLC plates by scraping the silica directly into scintillation vials, and quantified by liquid scintillation counting.

#### Sphingomyelinase assay

SMase activity was measured as previously described [28] with some modifications. Briefly, cell homogenates containing 50 µg protein were incubated in a final volume of 500 µL Tris-KCl buffer (25 mM KCl, 50 mM Tris pH 7.4 and 5 mM MgCl_2_) for the neutral sphingomyelinase assay or 500 µL sodium acetate buffer (50 mM sodium acetate pH 4.5) for the acid sphingomyelinase assay. The reactions were started by addition of 1 nmol C_6_–NBD-SM (ethanol stock mixed in the buffer) and incubated in the dark at 37 ^°^C for 20 minutes (nSMase) or 15 minutes (aSMase). Reactions were terminated by the addition of three volumes of chloroform: methanol (1:2; v/v). Lipids were extracted as mentioned earlier and separated by TLC using chloroform: methanol: 9.8 mM aqueous CaCl_2_ (60:35:8; v/v/v). NBD-labeled sphingolipids were identified using authentic standards by Fluor-S Max device and quantified using Image Quant program.

### Lipid extraction and LC/MS-MS analysis of sphingolipids

After exposure to OxPL as described above, cells were harvested with cold phosphate buffered saline. The pellet was re-suspended in a mixture of 100 µL distilled water and 750 µL chloroform: methanol (1:2; v/v) along with Cer/Sph Internal Standard Mixture I-LM6002 (Avanti Polar Lipids, Alabaster, USA). Samples were sonicated and incubated overnight at 48 ^°^C. Alkaline hydrolysis was carried out by adding 75 µL 1 M KOH in methanol, sonicated and incubated for 2 h at 37 ^°^C. Samples were then neutralized by adding 3 µL glacial acetic acid and lipids were partitioned in 3 mL chloroform: water (1:2; v/v). The upper aqueous layer was separated and the solvent was removed from the lower organic layer under N_2_. Lipid extracts were analyzed for ceramide species by LC/MS-MS [29]. Data are expressed as pmole of ceramide per mg of total protein.

## Results

### The oxidized phospholipids POVPC and PGPC are cytotoxic and induce apoptotic cell death in murine macrophages

**Figure 1:**
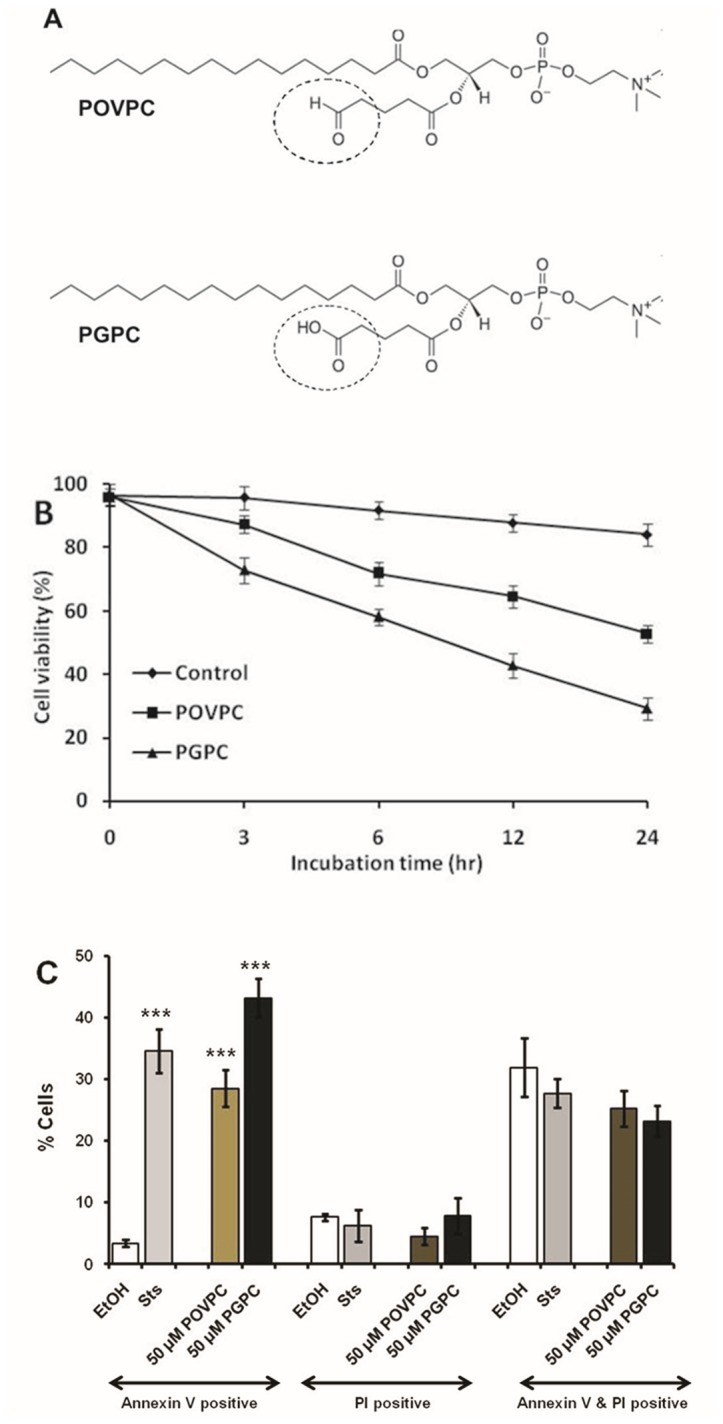
The oxidized phospholipids POVPC and PGPC are cytotoxic and induce apoptosis. (A) Chemical structures of POVPC and PGPC. Dotted circle represents the functional group at *sn-2* position. (B) RAW 264.7 cells were incubated with 50 µM POVPC and PGPC for indicated periods. Control cells were treated with 1% ethanol. Cell viability was determined by Vybrant® MTT assay kit. Results are expressed as a percentage of viable cell number in treated cells compared with that of untreated control cells. Data are means ± S.D., n = 8 in each group. (C) Cells were incubated with the stated concentrations of POVPC and PGPC for 4 h. The cells were analyzed for Alexa Fluor_488_-annexin V and propidium iodide fluorescence staining by Flow cytometry as described under “Experimental Procedures”. Results are represented as means ± S.D. Probabilities compared to control were determined by Student’s t-test (two-tailed, unpaired); ***p < 0.001,(n = 8 in each group).

To determine the effects of oxidized phospholipids, RAW 264.7 cells were treated with POVPC or PGPC (Figure 1A) and cell viability was measured. Figure 1B demonstrates reduced viability of macrophage cells upon incubation with either OxPL, in a time-dependent manner. Further, we treated the cells with OxPLs for 4 h and analyzed the externalization of phosphatidylserine in the outer leaflet of the plasma membrane, which is an early marker of apoptotic cell death. Both POVPC and PGPC increased the fraction of Annexin V-stained cells in a concentration-dependent manner indicating the induction of apoptotic cell death (Figure 1C). From cell staining with propidium iodide, it can be inferred that both OxPLs did not induce necrosis. The fraction of late apoptotic/ early necrotic cells was not increased by OxPL treatment either as compared to control cells which had only been incubated with EtOH. Exposure of the cells to PGPC or POVPC for more than 4 h (up to 24 h) led to similar but less reproducible results. We think that this phenomenon is due, at least in part, to the limited lifetime of the OxPLs in the incubation medium and inside the cells [26,30]. Collectively, the data suggest that both OxPLs are cytotoxic to cells, and that PGPC is a more potent inducer of programmed cell death as compared to POVPC.

### CerS expression and CerS activity in cultured murine macrophages

**Figure 2:**
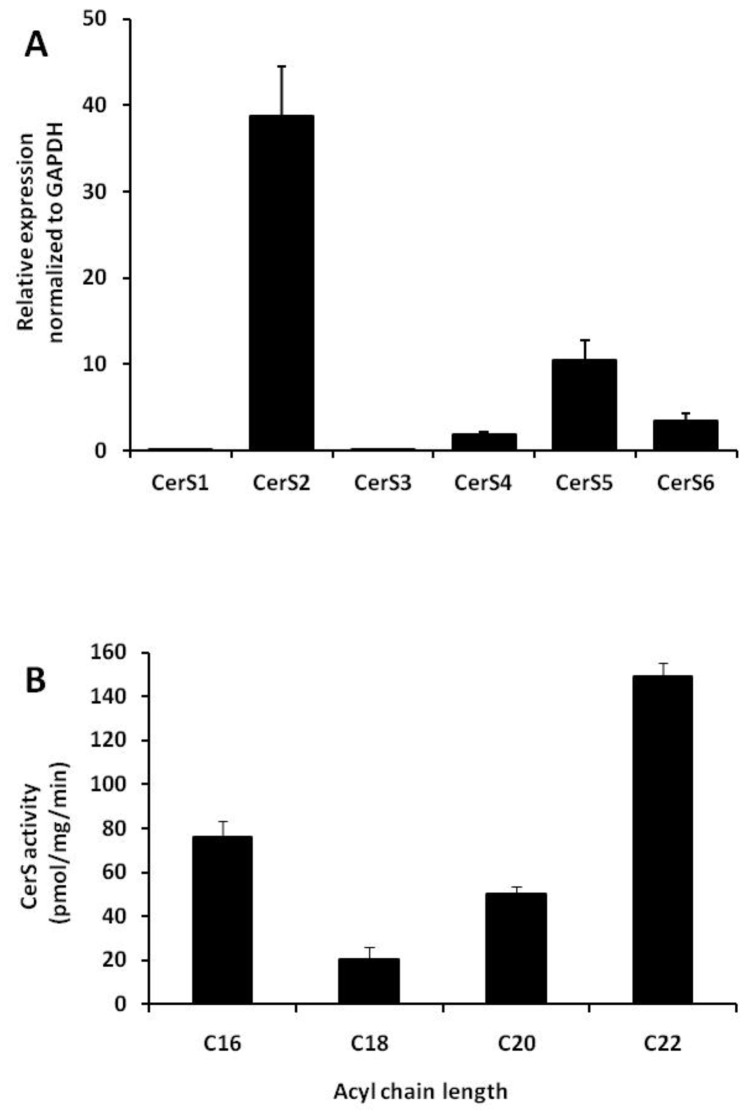
CerS expression and endogenous CerS activity in RAW 264.7 cells. (A) RAW 264.7 cells were harvested and cDNA was synthesized. CerS mRNA levels were measured by RT-qPCR as described under “Experimental Procedures”. The data are normalized to GAPDH mRNA expression and data are means ± S.E. for three independent experiments performed in triplicate. (B) CerS activity was assayed in cell homogenates using C_16_-CoA, C_18_-CoA, C_20_-CoA and C_22_-CoA substrates as described under “Experimental Procedure”. Results are means ± S.E. for four independent experiments.

Earlier studies demonstrated that the six CerS isoforms are differentially expressed in different tissues [16,31]. We assessed mRNA expression of CerS isoforms in cultured RAW 264.7 cells using RT-qPCR analysis. We found that CerS2 was the predominant isoform while CerS4, CerS5 and CerS6 were expressed at lower levels (Figure 2A). In contrast, CerS1 and CerS3 were virtually undetectable. These results demonstrate that CerS2 is the predominant CerS in RAW 264.7 macrophages.

We next examined CerS activity in cell homogenates using a range of acyl-CoAs as enzyme co-substrates. Quantification of the reaction product (dihydro) ceramide correlated with the mRNA expression levels. C_22:0_-Cer, the product of CerS2, was found to be abundantly synthesized comprising approximately 50% of total Cer. C_16:0_-Cer and C_20:0_-Cer comprised 26% and 17% of the total ceramide formed, respectively (Figure 2B). Thus, CerS2 was the most active CerS, with an activity of 149.2 ± 5.8 pmol/mg/min, while C_16_-Cer, which is generated by both CerS5 and CerS6, was synthesized at rates of 76.0 ± 7.2 pmol/mg/min. However, we could not detect the very long-chain products (C_26:0_-Cer) which are synthesized by CerS3. Thus, our data suggest that CerS2 is highly expressed and responsible for the generation of significant levels of ceramide in RAW 264.7 cells.

### Oxidized phospholipids induce changes in intracellular ceramide levels by influencing ceramide synthases in macrophage cells

**Figure 3:**
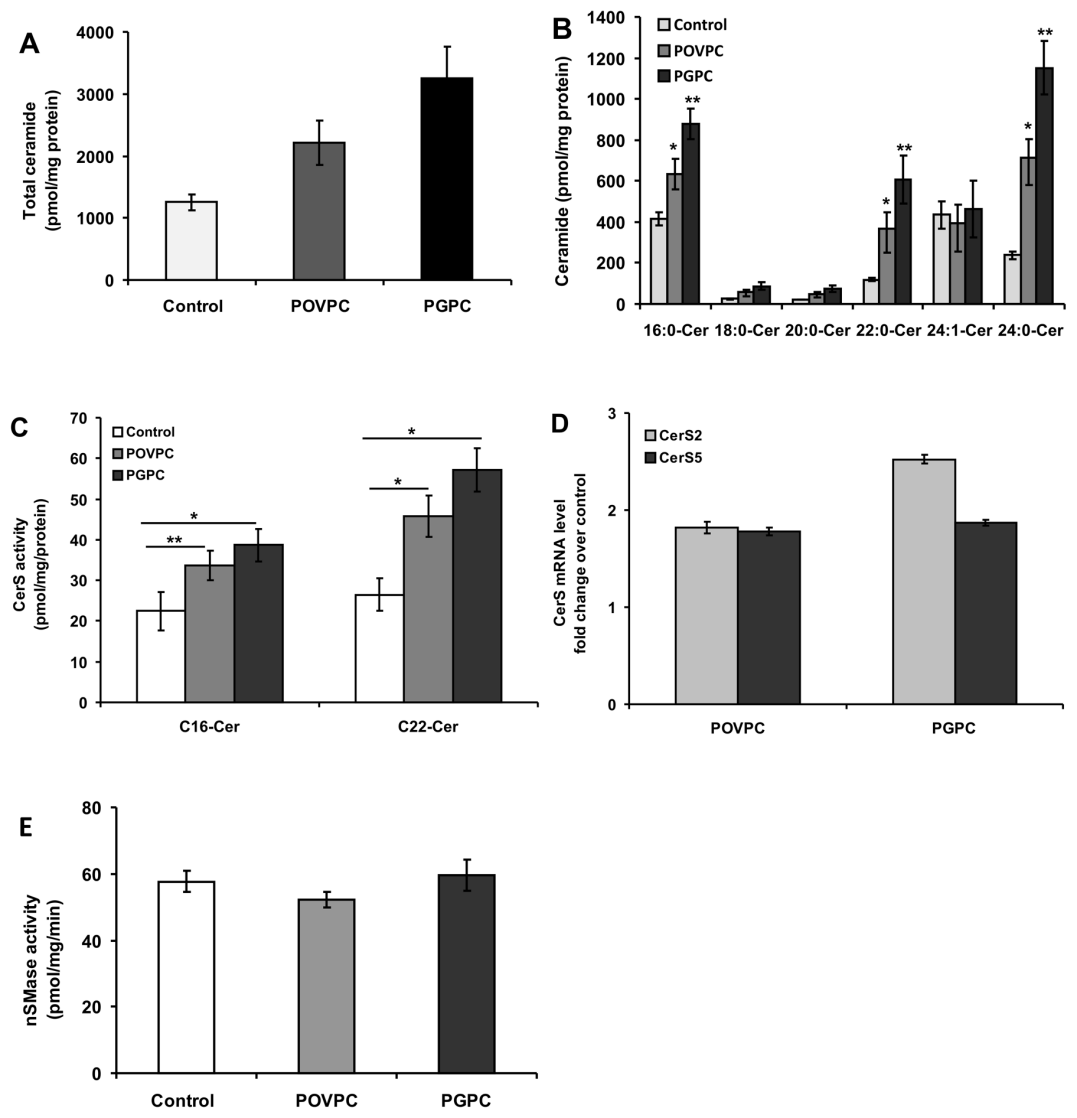
Influence of POVPC and PGPC on ceramide levels and CerS activation in RAW 264.7 cells. (A) Both POVPC and PGPC elevate ceramide generation in RAW 264.7 cells. Cells were stimulated for 24 h with respective OxPLs (50 µM) in parallel to ethanol treated control cells. Lipids were extracted and analyzed for ceramide levels by LC/MS-MS as described under “Experimental Procedure”. No probability. values are given for total ceramide levels because these levels are the sum of ceramide species with different acyl chain lengths. (B) Ceramide speciation was performed after OxPL treatment as above. The data are means ± S.E., *p<0.05, **p < 0.01 compared with control, n = 4. (C) After OxPL treatment cells were harvested and CerS activity in cell homogenates was measured as described earlier. Results are means ± S.E., *p < 0.01, **p < 0.05, of a typical experiment repeated four times with similar results. (D) CerS mRNA levels were measured by RT-qPCR after 24 h incubation with 50 µM POVPC or PGPC as described under “Experimental Procedures”. The data are normalized to GAPDH mRNA expression and data are means ± S.E. for four independent experiments performed in triplicate. (E) nSMase activity was measured in cell homogenates after exposure to OxPL as described under “Experimental Procedures”. The data are represented as means ± S.E., n = 4.

Previous studies demonstrated that intracellular ceramide levels in arterial smooth muscle cells (SMC) are elevated under the influence of OxPLs leading to apoptotic cell death [22]. In this study, we determined ceramide concentrations in untreated and OxPL-treated macrophages. Ceramide analysis by LC/MS/MS revealed a significant increase in total ceramide concentration in both POVPC- and PGPC-treated cells compared to untreated control cells (Figure 3A). Further analysis of ceramide species showed a significant increase in C_16:0_, C_22:0_ and C_24:0_-Cer (Figure 3B) in OxPL-treated cells. In contrast, elevation in C_18_-Cer and C_20_-Cer levels was statistically insignificant. In summary, this data indicates a selective effect of OxPLs on the levels of C_16_-and C_22:0_/C_24_-ceramides.

To find out whether activation of CerS or hydrolysis of sphingomyelin via sphingomyelinase is involved in OxPL-induced ceramide generation during long exposure times, we first examined the effects of both POVPC and PGPC on CerS activity in RAW 264.7 cells. Exposure of cells to 50 µM POVPC and 50 µM PGPC for 24 h resulted in a significant increase in CerS2 activity (45.8 ± 5.1 pmol.mg protein ^-1^ min^-1^ for POVPC and 57.2 ± 6.3 pmol.mg protein ^-1^ min^-1^ for PGPC) compared to control values (26.5 ± 3.9 pmol.mg protein ^-1^ min^-1^). CerS5/6 activities (33.6 ± 3.5 pmol.mg protein ^-1^ min^-1^ for POVPC and 38.7 ± 4.0 pmol.mg protein ^-1^ min^-1^ for PGPC) were also higher compared to controls (22.4 ± 4.7 pmol.mg protein ^-1^ min^-1^) (Figure 3C).

To determine the influence of OxPLs on CerS gene expression, we assessed mRNA levels in cells exposed to OxPLs. PGPC treatment led to a more than 2.5 fold increase of CerS2 mRNA over control while, POVPC led to a nearly 2 fold increase in CerS2 and CerS5 mRNA levels (Figure 3D).

We next measured sphingomyelinase activity in RAW 264.7 cells exposed to OxPL for 24 h. Neither change in neutral (nSMase) (Figure 3E) nor acid sphingomyelinase (aSMase) activities were observed (data not shown). Together, the data suggest that OxPL-induced ceramide generation in RAW 264.7 cells is likely to be due to the activation of distinct CerS isoforms. C_16_-Cer, has been shown to be the product of CerS5 and CerS6 activities, whereas C_22_-and C_24:0_-Cer species are synthesized by CerS2. Elevation of specific Cer species by OxPLs correlated well with OxPL-mediated stimulation of the particular CerS isoforms that catalyze the formation of the respective Cer species.

### Fumonisin B1 inhibits ceramide synthases and abrogates ceramide elevation

**Figure 4:**
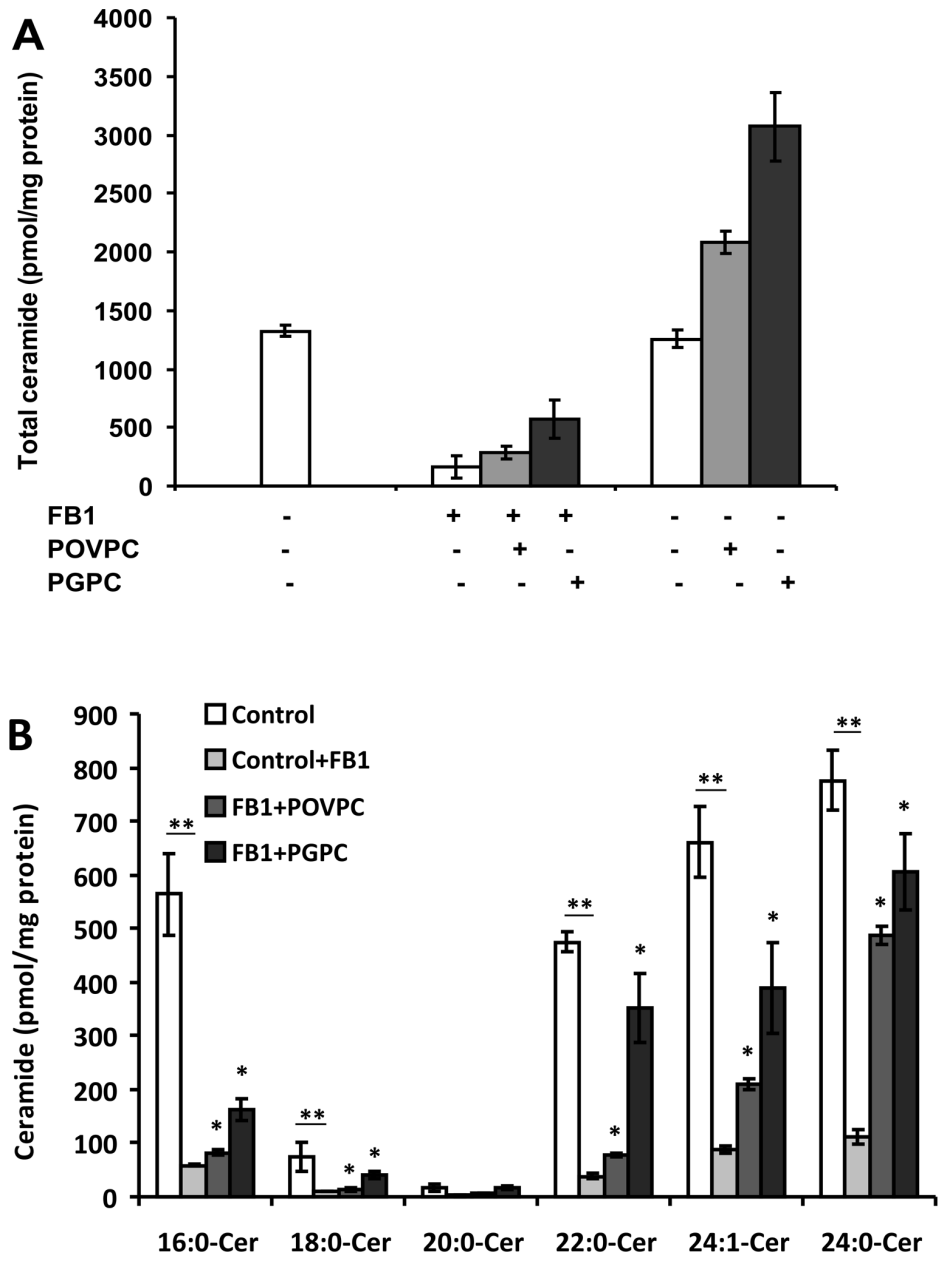
Effect of FB1 on OxPL induced CerS activation and ceramide levels. (A) Raw 264.7 cells were pre-incubated with FB1 (20 µM) for 2 h prior to the 24 h OxPL treatment. Lipids were extracted and ceramide levels were analyzed as described under “Experimental Procedure”. No probability values are given for total ceramide levels because these levels are the sum of ceramide species with different acyl chain lengths. (B) Ceramide species were analyzed as earlier. The data are means ± S.E., *p < 0.05, **p < 0.01 compared with control, n = 4.

FB1 is a potent and specific inhibitor of CerS. Thus, we examined the effect of FB1 on OxPL-induced Cer generation. Cells were pre-incubated with FB1 (20 µM) for 2 h and then exposed to OxPLs for 24 h. As expected, we found that the ceramide content of control cells was reduced significantly in the presence of FB1 (Figure 4A). It is noteworthy that even though FB1 abrogated ceramide accumulation in control cells, we could observe the stimulation of ceramide synthesis under the influence of OxPLs. Notably, both OxPL-induced ceramide synthase activation and ceramide levels were sensitive to FB1 (Figure 4B).

We then measured CerS activity under the influence of OxPLs with or without pretreatment with FB1. Indeed, OxPL-induced CerS activity and base-line enzyme activities were sensitive to FB1. It is noteworthy that even though FB1 abrogated CerS activity, we could still observe the difference in CerS activity under the influence of OxPLs compared to the enzyme activity in FB1-treated control cells (data not shown). Together, the results of these studies suggest that OxPLs-triggered CerS activation in macrophages is responsible for the elevation of ceramide levels.

### Lipid extracts from LDL and OxLDL elevate CerS activity in macrophage cells

**Figure 5:**
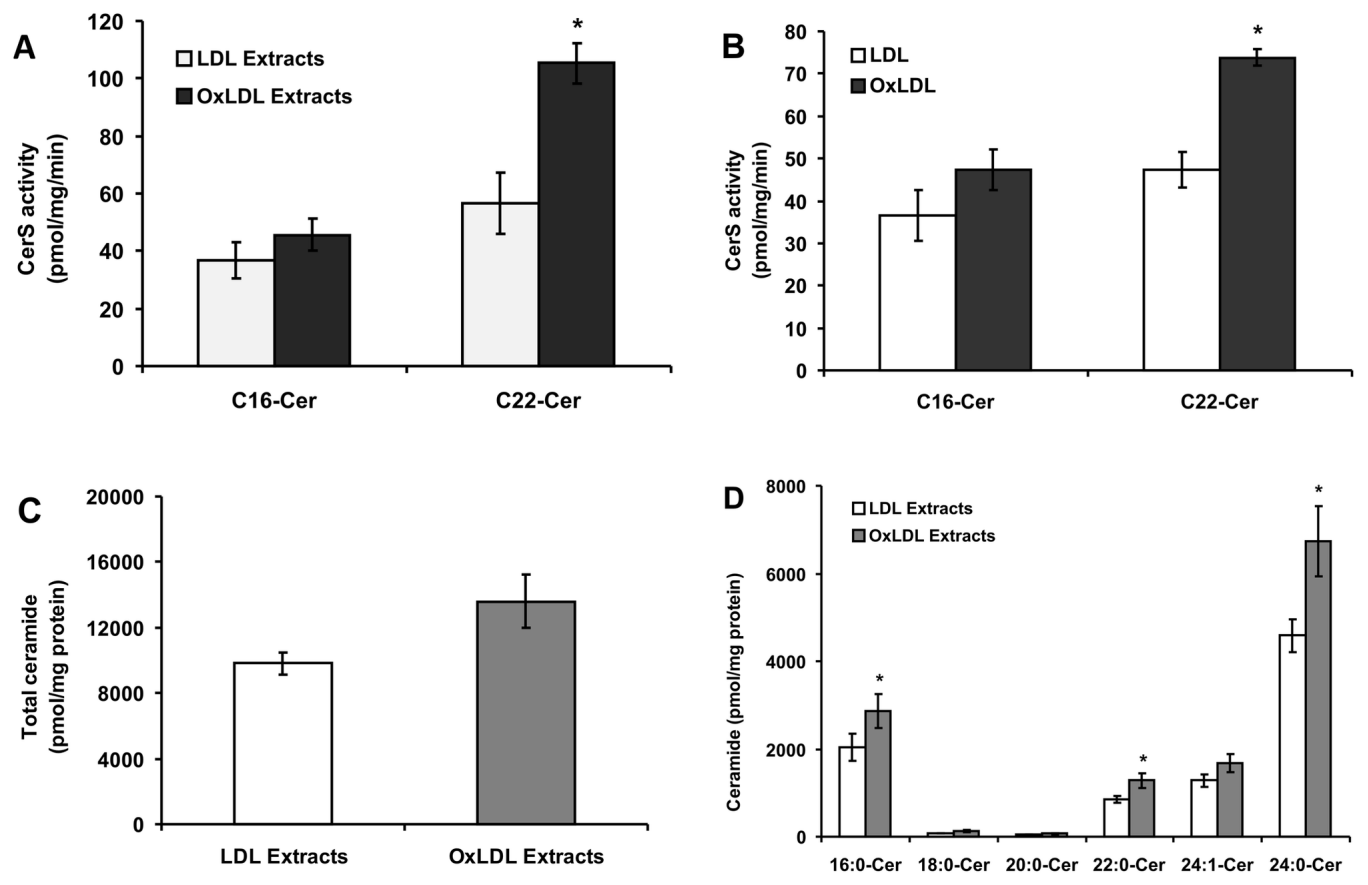
Effect of lipid extracts from LDL and OxLDL on CerS activity. (A) Oxidation of LDL was performed as described under “Experimental Procedure”. RAW 264.7 cells were stimulated with lipid extracts from native LDL and OxLDL (50 µg protein/mL respectively) for 24 h. CerS activity in cell homogenates was measured using C_16_-CoA and C_22_-CoA substrates. Results are means ± S.D., *p < 0.05, of a typical experiment repeated four times with similar results. (B) Cells were treated with intact LDL and OxLDL (50 µg protein/mL respectively) as described above for 24 h. CerS activity in cell homogenates was measured using C_16_-CoA and C_22_-CoA substrates. Results are means ± S.D. *p < 0.05, of a typical experiment repeated four times with similar results. (C) Cells were treated as described above and lipids were extracted and analyzed for ceramide content as described under “Experimental Procedure”. No probability values are given for total ceramide levels because these levels are the sum of ceramide species with different acyl chain lengths. (D) Ceramide species were analyzed after treatment with lipid extracts from native LDL and OxLDL as described earlier. The data are means ± S.E., *p < 0.05, n = 4.

It has been shown that POVPC and PGPC are present in OxLDL and contribute to the deleterious effects of OxLDL. To demonstrate a functional role of the lipid oxidation products in elevating ceramide levels, we treated macrophage cells with lipid extracts from LDL and OxLDL and examined changes in CerS activity. As shown in Figure 5A, lipid extracts from OxLDL significantly increased CerS2 activity after 24 h of treatment, whereas CerS5/6 activity was only slightly elevated compared to cells incubated with lipid extracts from native LDL. In addition, we analyzed CerS activity after exposing cells to intact LDL and OxLDL. As expected, intact OxLDL elevated CerS activity in a fashion that is similar to that of the pure POVPC and PGPC (Figure 5B). Lipid extracts from OxLDL induced an even stronger effect than the intact lipoprotein. These results indicate that lipid oxidation products such as POVPC and PGPC present in OxLDL are likely to contribute to the increased lipoprotein capacity of activating CerS enzymes.

We then analyzed ceramide levels in the cells treated with total lipid extracts from LDL and OxLDL. As expected, total ceramide content was elevated significantly in cells treated with the lipid extracts from OxLDL compared to cells treated with lipid extracts from native LDL (Figure 5C). In agreement with the data shown in Figure 5A, further analysis of ceramide species revealed a significant elevation in C_16_-, C_22_-, C_24:1_-and C_24:0_-Cer levels (Figure 5D). Taken together, the results suggest that the increase in ceramide content under the influence of total lipid extracts from OxLDL is likely due to activation of CerS enzymes by lipid oxidation products such as POVPC and PGPC present in OxLDL.

## Discussion

The present study demonstrates that POVPC, PGPC and OxLDL trigger ceramide accumulation in RAW 264.7 cells. These results support the assumption that the elevation in ceramide content is mediated by the OxPL-induced activation of CerS that are key enzymes of ceramide biosynthesis. Both POVPC and PGPC activated particular CerS isoforms catalyzing the synthesis of ceramide species containing fatty acyl-residues with specific chain lengths. We demonstrate that PGPC is a potent activator of CerS2 whereas CerS5/6 is activated to a lesser extent. Since both OxPLs and OxLDL show the same effect on Cer formation, we conclude that POVPC and PGPC are active components of the modified lipoproteins and contribute to its capacity of stimulating CerS synthesis. These studies highlight the induction of ceramide generation by oxidized phospholipids and the activation of specific enzymes involved in ceramide biosynthesis.

Under the influence of various stress stimuli, ceramide can be generated by three major pathways, namely, sphingomyelin hydrolysis, the salvage pathway and *de novo* synthesis. Depending on stimulus or cell type, single or multiple pathways may be activated [32,33]. In earlier studies, a correlation was established between apoptotic signaling and the fast POVPC- and PGPC-induced activation of aSMase in vascular smooth muscle cells [22], and RAW 264.7 macrophages [26]. The present study suggests that POVPC and PGPC also activate CerS, but after long incubation times, thereby leading to accumulation of distinct ceramide species. The suppression of OxPL-induced ceramide formation by FB1 supports the assumption that CerS catalyze the formation of these lipids. PGPC is a more potent activator of CerS enzymes than POVPC. This effect is due to the tiny structural difference between both OxPLs (Figure 1A, dotted circles) which determines their chemical reactivity and cellular localization. POVPC, containing a reactive aldehyde group at the *sn-*2 position can form covalent Schiff-bases with the free amino groups of proteins and amino-phospholipids. Thus, it is retained in the plasma membrane for longer periods where it may modify SMase [34]. In contrast, PGPC contains a ω-carboxylic group and cannot form covalent bonds with other biomolecules. As a consequence, it is rapidly internalized [35] and can influence the activities of enzymes (such as CerS) inside the cells. Fluorescence microscopy studies revealed that labeled PGPC becomes enriched in intracellular membranes. As a consequence, the OxPL can modulate the activities of intracellular membrane proteins (e.g. CerS localized to the ER) either indirectly by affecting membrane lipid organization or by direct lipid-protein interaction. Thus it can be speculated that in addition to transcriptional effects, these enzymes are activated by OxPLs in the above defined fashion.

Both POVPC and PGPC have been identified as major biologically active lipids present in oxidatively modified LDL [36]. It has been shown that the respective lipids [21,22], as well as the entire OxLDL particle, trigger an early activation of sphingomyelinase and ceramide formation to propagate their biological effects in various cells [37–39]. However, information was lacking about the capacity of OxLDL and its oxidized phospholipids to activate CerS which utilize sphingosine and sphinganine for Cer formation in the recycling pathway and *de novo* sphingolipid synthesis, respectively. Here, we show that, over a long incubation period, POVPC, PGPC, intact OxLDL and total lipid extracts from OxLDL activate CerS in a similar fashion. Obviously, POVPC and PGPC present in the extracts of OxLDL are likely to contribute to the increased activation of CerS enzymes in RAW 264.7 cells.

Activation of distinct CerS isoforms has been shown in different cell types under various stress conditions [12,19,40–42]. Our studies demonstrate that POVPC and PGPC activate specific sub-sets of CerS isoforms in RAW 264.7 cells to a different extent. The profiles of ceramide species that accumulate under the influence of both OxPLs are consistent with the observed activation of CerS2 and CerS5/6, which have been shown to be responsible for the generation of C_22:0_-, C_24:0_-, and C_16:0_-ceramide, respectively. It will be the aim of future studies to determine the biophysical and biochemical effects of the formed ceramide on the (sub) cellular membranes and their consequences for cell physiology.

Both OxPLs closely mimic the toxic properties of OxLDL in cultured vascular cells. Chronic exposure to µM concentrations of POVPC and PGPC induce apoptosis in cultured SMC and macrophages. Lipid-induced cell death is associated with the formation of ceramide which is considered an apoptotic messenger propagating physical, chemical and biochemical stress. However, the mechanisms of the ceramide response to POVPC and PGPC seem to be different. Whereas POVPC is a more efficient activator of aSMase at short exposure times, PGPC elicits a much more pronounced ceramide response by the FB1-sensitive CerS activity after long incubation times. Thus, initiation of ceramide formation by OxPLs is biphasic, showing an initial peak of aSMase activity under the influence of POVPC and a late persistent activation of CerS by PGPC. Thus, it can be speculated that OxLDL containing both compounds efficiently and persistently activates ceramide formation in the cells of the vascular wall. It remains to be clarified whether and to what extent the OxPL-induced CerS- and Cer-associated stress response in vascular cells is relevant to the patho-biochemistry of atherosclerosis. If this were the case, it would worth studying the biophysical and biochemical mechanisms of CerS-catalyzed ceramide formation and its consequences for programmed cell death on the cellular and molecular level.

In summary, the present study demonstrates that the OxLDL components POVPC and PGPC trigger the activation of ceramide synthases, but not sphingomyelinase, after prolonged incubation resulting in accumulation of ceramide in RAW 264.7 cells. PGPC is a more potent inducer of ceramide generation compared to the structurally similar but chemically different POVPC.
